# Profiles and Temporal Dynamics of Personality-Related Language in a 16-year Professional Email Archive: A Subject-Specific Study (2008-2024)

**DOI:** 10.17505/jpor.2026.29457

**Published:** 2026-08-24

**Authors:** Krzysztof Rybinski

**Affiliations:** Department of Business and International Relations, Vistula University, Stoklosy 3 Str., 02-787 Warsaw, Poland. Emails: rybinski@rybinski.eu, k.rybinski@vistula.edu.pl. Phone: +48 609 56 29 21. https://orcid.org/0000-0002-4604-7993

**Keywords:** person-oriented research, subject-specific study, within-person, personality-related language, longitudinal analysis, Self-Determination Theory, Big Five, Psychological Well-Being

## Abstract

This article reports a subject-specific, within-person self-study of personality-related language in one participant-author’s predominantly professional email archive (2008-2024; *n* = 25,780). The aim is not between-person prediction or human-parity benchmarking, but idiographic description of one person’s profile and temporal dynamics across Self-Determination Theory, the Big Five, and Psychological Well-Being. Email texts were coded with two LLM-assisted workflows – prompted GPT-3.5 ratings and a GPT-4.1 teacher distilled into compact multilingual transformer students – with cross-model comparisons treated as robustness checks. Email-level labels were aggregated into monthly balance indices on a common [-100, 100] scale. Within-person profile correspondence with contemporaneous self-report reference profiles was examined on POMP-aligned scales, and monthly shifts across pre-specified major personal and occupational periods were estimated using HAC-robust regressions with FDR control. Across frameworks, the archive yielded interpretable subject-specific patterns: email-derived profiles partly approximated the self-report reference shape while also reflecting the professional register, and month-level indices shifted in theory-coherent ways across salient periods. The findings support de-identified digital traces as state-sensitive adjuncts for person-oriented research on one individual’s functioning over time. The results do not warrant between-person inference, and self-reports remain the primary reference for trait baselines.

## Introduction

Digital traces such as emails can capture how one person’s language varies across contexts and over time. In person-oriented research, that possibility is valuable when the aim is to understand an individual configuration of functioning rather than to estimate population averages. In Lundh’s (2015) formulation, this places the study on the individual-focused and pattern-focused side of psychological inquiry, and Nilsson’s (2024) more recent discussion of person-sensitive measurement underscores why designs that prioritize individual interpretability remain methodologically important. Recent progress in generative AI, especially Large Language Models (LLMs), has expanded the feasibility of reading large naturalistic text archives through theory-guided coding schemes.

The person-oriented point of departure is that the individual person, or the person’s pattern of functioning, is the primary unit of interpretation. The archive is therefore not used to estimate a population model or to rank people against one another. It is used as a repeated record of how one person expresses motivational, trait-related, and well-being-relevant language across time and circumstances. This is close to subject-specific and single-case traditions, but it differs from many diary or ecological momentary assessment studies because the observations are naturally occurring digital traces rather than researcher-designed repeated items. It also differs from much computational personality work because the main question is not cross-person model accuracy; LLMs are used here as theory-guided coders to assist an idiographic reading.

Evidence on LLM performance is mixed. Early evaluations found that ChatGPT underperformed in comparison with earlier Natural Language Processing (NLP) systems on Big Five classification (Amin et al., 2023), while embedding-based pipelines using LLM representations or distilled models have sometimes performed better in domain-specific settings (Maharjan et al., 2025; Hu et al., 2024). Much of that literature, however, is benchmark-oriented and only indirectly addresses whether one person’s language archive can be used to describe that person’s profile and changing circumstances over time.

The present study is therefore framed as a subject-specific, within-person analysis of one person’s predominantly professional email archive. The goal is to examine whether deidentified email language yields interpretable profiles and temporal shifts when read through three established psychological frameworks and anchored to contemporaneous self-report benchmarks. The contribution is idiographic: we ask what can be learned about one individual’s configurations and transitions, not how well a model ranks people against each other. In this sense, the study follows Molenaar’s (2015) argument that subject-specific analysis is not peripheral to person-oriented research but one of its most direct methodological expressions when intraindividual processes are nonergodic.

All emails were coded under three complementary frameworks: Self Determination Theory (SDT), the Five Factor Model (Big Five), and Psychological Well Being (PWB). SDT posits that motivation and adjustment hinge on the fulfilment of three basic needs: Competence, Autonomy, Relatedness (Deci & Ryan, 2000; Ryan & Deci, 2002). Big Five organizes personality into Neuroticism, Extraversion, Openness, Agreeableness, and Conscientiousness (McCrae & Costa, 1987; John & Srivastava, 1999). PWB conceptualizes eudaimonic functioning across six dimensions: Autonomy, Environmental Mastery, Personal Growth, Positive Relations with Others, Purpose in Life, and Self-Acceptance (Ryff, 1989; Ryff & Keyes, 1995). Together, these schemes allow convergent, theory specific readings of the same naturalistic texts while preserving parsimony (three-four label states per construct) and comparability across frameworks.

This design addresses three gaps relevant to person-oriented work with digital traces. First, cross-theory studies are rare: most prior work focuses on one construct family at a time, whereas individual functioning is better described as a configuration spanning broad traits, motivational needs, and well-being dimensions. Second, longitudinal subject-specific analyses remain uncommon, especially in archives that extend across major occupational and personal periods. Recent work on multiple single cases shows that dynamic patterns and contextual information can reveal transitions and resilience that are easily missed if within-person irregularities are treated merely as statistical nuisance (Olthof et al., 2024). Third, model comparisons are often treated as the main contribution; here they serve a secondary role as robustness checks around an idiographic interpretation.

These questions are examined in a within-person corpus of one person’s email archive using two LLM-assisted workflows (prompted GPT-3.5 and a GPT-4.1-based teacher-student pipeline), eleven model instances, and three theorylinked coding schemes. The methodological comparison is retained only to test whether the idiographic profile and temporal pattern are robust across plausible coding workflows; it is not the primary contribution of the article.

Two research questions are posed:

- RQ1 (within-person profile correspondence; Study 1). Within this subject-specific case, to what extent do emailderived indices approximate the shape of contemporaneous self-report reference profiles for SDT, Big Five, and PWB?
- RQ2 (within-person temporal dynamics; Study 2). Within this subject-specific case, how do monthly indices shift across pre-specified major personal and occupational periods in theory-coherent ways?

Again, it should be noted that the aim is idiographic interpretation of one person’s language archive in a mostly professional register, not between-person inference, human-parity testing, or a cross-model leaderboard.

The three frameworks are used as complementary languages for describing one person’s functioning, not as interchangeable measures of the same latent variable. SDT highlights perceived need support and need frustration; the Big Five provide a broad vocabulary for trait-related behavioral tendencies; and PWB describes eudaimonic functioning. At the same time, these constructs are not assumed to be continuously visible in every email. Personality expression in everyday behavior depends on situations, roles, affordances, and the person’s choice to disclose or withhold particular feelings and concerns (Mischel & Shoda, 1995; Fleeson, 2001; Funder, 2006). Professional email is especially constrained by task, recipient, institutional role, and politeness norms. Consequently, the email-derived scores are interpreted as personality-related language in a professional communicative context, not as direct measurements of latent traits, needs, or well-being.

## Materials and Methods

### Data

The data used in this research are described below. A brief ethics and data-availability statement is included here, with fuller governance details in Appendix A. This article reports a subject-specific self-study in which the sole author was also the sole participant and source of archival data. On 3 March 2026, the Ethics and Anti-Discrimination Committee of Vistula University approved the project from an ethical standpoint for publication. The signed approval form did not state a reference number. The participant-author provided written informed consent for archive retrieval, de-identification, analysis, and publication of aggregate findings.

#### Dataset of 25,780 emails written by the participant-author over sixteen years

Using the Google Gmail API, 25,780 emails (each >= 10 words) authored by the participant-author between January 2008 and March 2024 were collected. The dataset was deidentified: email addresses were hashed, quoted/forwarded text from others was excluded, and all reporting is limited to aggregate outputs and generalized contextual descriptions. No verbatim email text is reproduced in the article or Supplementary materials.

Email activity varied substantially over time - from near zero in late 2016 to > 500 emails/month in 2010 - 2011 and the number of unique recipients fluctuated from very few to ≈ 200/month. The average monthly email length (excluding appended quotations) was 66 words, with considerable month-to-month variability. For psychological construct extraction, only the first 300 words of each email were analyzed; as a result, 3.8% of emails were truncated. Messages were written primarily in English and Polish, with some in Russian or in mixed Polish-English; the LLMs used in this study natively support multilingual input. The majority of messages concerned the participant-author’s professional activities.

#### Psychometric tests

As part of this subject-specific self-study, the participantauthor completed self-report measures aligned to the psychological theories described above. Table 1 reports the available raw scores, possible score ranges, and profile scores used for the within-person reference profiles. The SDT scores are based on the Basic Psychological Need Satisfaction and Frustration Scale (BPNSFS; Van der Kaap-Deeder et al.. 2020), with satisfaction and frustration components reported separately and a need-balance score used in profile displays. The Big Five reference profile is based on the OpenPsychometrics (https://openpsychometrics.org/tests/IPIP-BFFM/) implementation of the 50-item IPIP Big-Five Factor Markers; because the archived output retained percentile scores rather than item-level responses, the Big Five profile is treated as a percentile reference rather than as a raw-score/POMP profile. PWB is based on the Ryff Psychological Well-Being forms distributed through SPARQtools (https://sparqtools.org/mobility-measure/psychological-wellbeing-scale/).

**Table 1:** Self-report reference profiles of the participant-author, with score ranges and profile transformations.

Framework	Construct	Instrument / reference	Reported score	Range and profile transformation
SDT	Autonomy	BPNSFS (Van der Kaap-Deeder et al., 2020)	Satisfaction = 19; frustration = 7	Each component 4-20; profile balance = POMP(satisfaction) - POMP(frustration) = 75.0
SDT	Relatedness	BPNSFS (Van der Kaap-Deeder et al., 2020)	Satisfaction = 10; frustration = 13	Each component 4-20; profile balance = -18.8
SDT	Competence	BPNSFS (Van der Kaap-Deeder et al., 2020)	Satisfaction = 14; frustration = 13	Each component 4-20; profile balance = 6.3
Big Five	Extraversion	IPIP Big-Five Factor Markers / OpenPsychometrics (Goldberg, 1992; Goldberg et al., 2006)	21st percentile	Percentile range 0-100; plotted score = -58
Big Five	Neuroticism	IPIP Big-Five Factor Markers / OpenPsychometrics (Goldberg, 1992; Goldberg et al., 2006)	Emotional stability = 98th percentile	Percentile range 0-100; reversealigned Neuroticism plotted score = -96
Big Five	Agreeableness	IPIP Big-Five Factor Markers / OpenPsychometrics (Goldberg, 1992; Goldberg et al., 2006)	35th percentile	Percentile range 0-100; plotted score = -30
Big Five	Conscientiousness	IPIP Big-Five Factor Markers / OpenPsychometrics (Goldberg, 1992; Goldberg et al., 2006)	91st percentile	Percentile range 0-100; plotted score = 82
Big Five	Openness	IPIP Big-Five Factor Markers / OpenPsychometrics (Goldberg, 1992; Goldberg et al., 2006)	Intellect/Imagination = 70th percentile	Percentile range 0-100; plotted score = 40
PWB	Autonomy	Ryff 18-item and 42-item forms / SPARQtools (Ryff, 1989; Ryff & Keyes, 1995)	18-item = 16; 42-item = 44	Ranges 3-21 and 7-49; POMP = 72.2 and 88.1
PWB	Environmental Mastery	Ryff 18-item and 42-item forms / SPARQtools (Ryff, 1989; Ryff & Keyes, 1995)	18-item = 16; 42-item = 45	Ranges 3-21 and 7-49; POMP = 72.2 and 90.5
PWB	Personal Growth	Ryff 18-item and 42-item forms / SPARQtools (Ryff, 1989; Ryff & Keyes, 1995)	18-item = 21; 42-item = 48	Ranges 3-21 and 7-49; POMP = 100.0 and 97.6
PWB	Positive Relations with Others	Ryff 18-item and 42-item forms / SPARQtools (Ryff, 1989; Ryff & Keyes, 1995)	18-item = 7; 42-item = 25	Ranges 3-21 and 7-49; POMP = 22.2 and 42.9
PWB	Purpose in Life	Ryff 18-item and 42-item forms / SPARQtools (Ryff, 1989; Ryff & Keyes, 1995)	18-item = 21; 42-item = 43	Ranges 3-21 and 7-49; POMP = 100.0 and 85.7
PWB	Self-Acceptance	Ryff 18-item and 42-item forms / SPARQtools (Ryff, 1989; Ryff & Keyes, 1995)	18-item = 20; 42-item = 45	Ranges 3-21 and 7-49; POMP = 94.4 and 90.5

*Note*. BPNSFS = Basic Psychological Need Satisfaction and Frustration Scale. SDT profile values use satisfaction POMP minus frustration POMP for each need. Big Five values are percentile outputs from the OpenPsychometrics 50-item IPIP Big-Five Factor Markers; item-level responses were not retained, so Big Five raw/POMP scores are not reported. PWB values are raw subscale scores from Ryff 18-item and 42-item forms; POMP is reported to show the range-based transformation used for profile comparisons. POMP = 100 x [(observed score - minimum possible score) / (maximum possible score - minimum possible score)]. The self-report measures provide a pragmatic within-person reference point rather than an external gold standard. The comparisons focus on profile shape and relative emphasis across dimensions, not strict score equivalence.

#### Coding of emails in terms of the three frameworks

All emails were coded under three complementary frameworks: Self-Determination Theory (SDT), the Five-Factor Model (Big Five), and Psychological Well-Being (PWB). For illustrative privacy-safe examples of construct coding, see Table 2.

**Table 2:** Illustrative privacy-safe examples of construct coding.

Framework	Construct	Positive / high / enhancing cue	Negative / low / struggle cue
SDT	Autonomy	Choosing a course of action, setting priorities, or explaining a self-endorsed decision.	Pressure, lack of discretion, or frustration about being constrained by others.	
SDT	Competence	Successful completion, mastery, problem solving, or confidence in handling a task.	Failure, difficulty, inability to complete work, or concern about insufficient skill or capacity.
SDT	Relatedness	Warm coordination, appreciation, mutual support, or maintaining connection with others.	Conflict, isolation, rejection, or relationship strain.
Big Five	Openness	New ideas, intellectual exploration, creative alternatives, or curiosity.	Preference for routine, resistance to novelty, or narrowed attention to standard procedures.
Big Five	Conscientiousness	Planning, deadlines, responsibility, follow-through, and organized task management.	Disorganization, missed obligations, or weak follow-through.
Big Five	Extraversion	Social initiative, energetic outreach, persuasion, or active engagement with recipients.	Withdrawal, minimal social initiative, or low expressed energy in communication.
Big Five	Agreeableness	Cooperation, tact, gratitude, compromise, or concern for another person.	Criticism, antagonism, mistrust, or unwillingness to accommodate.
Big Five	Neuroticism	Anxiety, irritation, worry, perceived threat, or emotionally charged problem framing.	Calmness, emotional stability, or neutral problem handling without distress language.
PWB	Autonomy	Self-directed stance, principled choice, or independence in judgment.	Dependence on external approval or difficulty acting according to one’s own view.
PWB	Environmental Mastery	Effective organization of work/life demands or control of practical circumstances.	Being overwhelmed by circumstances or unable to manage demands.
PWB	Personal Growth	Learning, development, improvement, or movement toward new capacities.	Stagnation, blocked development, or frustration about lack of progress.
PWB	Positive Relations with Others	Trust, warmth, mutuality, and constructive interpersonal engagement.	Interpersonal strain, low trust, or lack of supportive connection.
PWB	Purpose in Life	Clear goals, direction, meaning, and commitment to valued aims.	Lack of direction, emptiness, or uncertainty about purpose.
PWB	Self-Acceptance	Balanced acknowledgment of strengths and weaknesses or self-accepting reflection.	Self-criticism, regret, shame, or rejection of aspects of the self.

*Note*. Entries are privacy-safe generic coding cues, not verbatim excerpts, paraphrases, or reconstructions of particular emails; they summarize the coding logic applied to the actual archive without reproducing raw email text.

SDT posits that motivation and adjustment hinge on the fulfilment of three basic needs: Competence, Autonomy, Relatedness (Deci & Ryan, 2000; Ryan & Deci, 2002). At the email level, each need received one of four labels: *Present* (clear evidence of the need; e.g., successful project → *Competence*), *Struggle* (difficulty or threat to the need; e.g., frustration about decision latitude → *Autonomy*), *Absent* (no discernible signal beyond routine content), or *None* (insufficient information, e.g., one-line confirmations).

Big Five organizes personality into Neuroticism, Extraversion, Openness, Agreeableness, and Conscientiousness (McCrae & Costa, 1987; John & Srivastava, 1999). For each email and trait, coders (models) assigned *High, Low*, or *None*, reflecting clear expression (*High*; e.g., proposing novel approaches → *Openness*), attenuated expression (*Low*), or insufficient content (*None*).

PWB conceptualizes eudaimonic functioning across six dimensions: *Autonomy, Environmental Mastery, Personal Growth, Positive Relations with Others, Purpose in Life*, and *Self-Acceptance* (Ryff, 1989; Ryff & Keyes, 1995). Each dimension was labeled *Enhancing* (active progress or achievement; e.g., effective life/work structuring → Environmental Mastery), *Maintaining* (stable, adequate functioning), *Struggling* (reported obstacles or dissatisfaction; e.g., unmet goals → Personal Growth), or *Not Applicable* (insufficient content, e.g., transactional scheduling).

Together, these schemes allow convergent, theory-specific readings of the same naturalistic texts while preserving parsimony (three-four label states per construct) and comparability across frameworks.

#### Contextual variables used for idiographic interpretation

Several contextual variables were assembled to aid subject-specific interpretation of month-level shifts. They were defined from dated administrative and personal records before inspection of the language trajectories and then held fixed across all model families. These descriptors are not treated as population predictors; they summarize broad personal, occupational, and environmental periods that structure this one archive.

Descriptions of variables (generalized labels are used in the manuscript to reduce identifiability):

- work-activity index: combined monthly work-related receipts normalized to the person’s period-wide average.
- personal expenditure index: monthly card expenditures normalized to [0,1].
- long-distance work assignment: dummy variable set to 1 for months with an extended work assignment abroad.
- regional work assignment: dummy variable set to 1 for months with a shorter-distance work assignment abroad.
- acute family/external stress period: variable set to 1 in the month combining close-family stress with major external stress; 0.75 in the following month and 0.5 in the month after that.
- second acute family stress period: variable set to 1 in the month of another close-family stress event; 0.75 in the following month and 0.5 in the month after that.
- family conflict period: dummy variable set to 1 for months with an emotionally demanding family conflict.
- major occupational transition A: dummy variable set to 1 for months with a high-intensity senior professional role whose demands differed sharply from the usual baseline.
- major occupational transition B: dummy variable set to 1 for months with a senior executive role in a technology-focused organization.
- high-visibility public activity period: dummy variable set to 1 for months with unusually public-facing professional activity.
- covid lockdown: dummy variable set to 1 for months with strict government lockdown measures.
- recipient diversity: number of different email recipients each month, normalized to [0,1].
- average email length: average number of words in emails sent each month, normalized to [0,1].

A minimal contextual summary and the generalized coding logic for these variables are provided in Appendix B. To reduce re-identification risk, several originally distinct realworld events were collapsed into broader categories, and labels were fixed before the language outputs were inspected. The contextual variables are used only to interpret withinperson patterns, not for between-person inference.

### LLMs, smaller transformer models and AI agents

In this study, LLMs are used as theory-guided coders of naturalistic text, and smaller transformer models are used as scalable students of the strongest teacher labels. Multiple model variants are retained mainly as robustness checks on whether the within-person profile and temporal dynamics are stable across plausible coding workflows.

Conceptually, a prompted LLM rating is analogous to asking a trained human coder to read one email and choose a label from a codebook. Zero-shot prompting means that the model receives only the coding rules. Few-shot prompting means that the same rules are accompanied by a small number of labeled examples. Fine-tuning means that a smaller text model is trained on many labeled examples so that it can reproduce the coding rule at lower cost. These elements – prompting, examples, fine-tuning, cross-validation, and comparison with alternative model families – are standard or increasingly common in computational text analysis. The less standard element in this study is the combination of three theory-based codebooks, a reflective GPT-4.1 teacher, and a long single-person archive for person-oriented interpretation rather than benchmark classification.

The corpus was scored in four passes designed to balance simplicity, robustness, and cost:

- First pass (zero shot). All emails were rated with GPT 3.5 Turbo using only carefully written instructions - no examples - for each theory.
- Second pass (few shot; “rater”). The same model (GPT 3.5 Turbo) was prompted with the original instructions plus seven human labeled examples (few shot learning). This pass is the rater.
- Third pass (few shot; “evaluator”). Again GPT 3.5 Turbo, with the same instructions but a different set of seven examples. This pass is the evaluator, and it provides an independent comparison to the rater.
- Fourth pass (teacher-student distillation). A GPT-4.1based agentic teacher labeled a random sample of 2,000 emails. Those labels were then used to finetune four smaller multilingual transformer architectures (“microsoft/mdeberta-v3-base”, “bert-basemultilingual-cased”, “distilbert-base-multilingualcased”, and “xlm-roberta-base”) in both separate-encoder and shared-encoder variants.

After fine-tuning, all emails were rated by the eight student models (four architectures x two training variants).

The rationale is practical rather than competitive: a more careful but expensive teacher can label a subset, while cheaper students scale the same coding logic to the full corpus. In this article the teacher-student workflow is retained only to test whether the subject-specific patterns remain stable across alternative coding strategies (Hu et al., 2024; Pangakis & Wolken, 2024).

**Figure 1:**
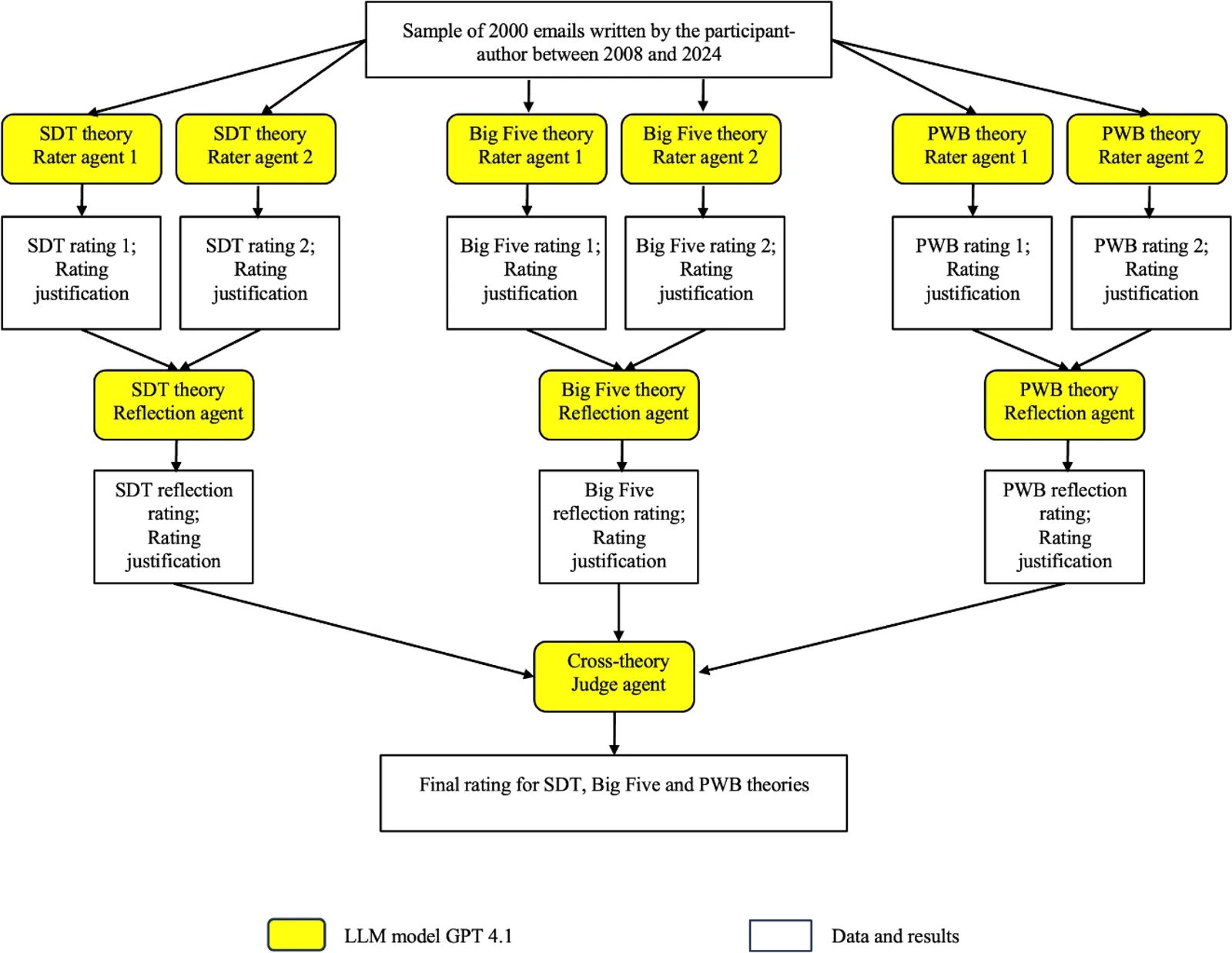
AI agentic structure.

Figure 1 summarizes the agentic teacher. For each sampled email and theory, two GPT-4.1 raters first assign labels, a reflection agent reconciles them, and a cross-theory judge checks whether the three theory-specific outputs are coherent. This staged teacher was used only on the training subset; the resulting labels then supervised the student models.

A condensed summary of prompts, model families, and fine-tuning settings is provided in Appendix D. Full prompts and implementation files are retained outside the article to keep the supplement concise.

### Study 1 - within-person profile correspondence vs. self-report reference profiles (RQ1)

To address RQ1, email-based ratings are compared with the self-report reference profiles reported in Table 1. Because the questionnaires were administered in 2024, the corpus of emails is restricted to 2022-2024 (*n* = 1,305) to ensure temporal proximity to the self-reports. Each email is machine-labeled on the relevant dimensions of the three theories. For the Big Five, ‘High’ counts as a positive signal and ‘Low’ as a negative signal; ‘None’ is ignored. An analogous rule is applied to SDT and PWB, with neutral states (e.g., ‘Maintaining’) excluded from the index. For each construct, a balance index is computed on a common [-100, 100] scale: (positives - negatives) / (positives + negatives), stabilized with a Jeffreys prior to mitigate small-denominator volatility.

To anchor the visual comparison (radar charts), a self-report reference profile is plotted on the same [-100, 100] axis. For the Big Five, the available OpenPsychometrics/IPIP output consisted of percentile scores; these percentiles are mapped linearly so that the 50th percentile = 0, values above map to > 0, and values below to < 0. Emotional Stability is reverse-aligned to Neuroticism. For SDT, satisfaction and frustration subscales are first converted to Percent of Maximum Possible (POMP; Cohen et al., 1999), and then the need-balance score is computed as satisfaction POMP minus frustration POMP. For PWB, the 18-item and 42-item Ryff subscales are converted to POMP before recentering/rescaling to [-100, 100]. This range-based transformation is preferable here to *z*-standardization because the aim is to compare within-case profile shape on a common interpretable scale rather than to express relative standing in sample-specific standard deviations (Moeller, 2025). This yields shapecomparable profiles between self-report and email-derived indices while avoiding stronger measurement claims than the design can support.

### Study 2 - within-person temporal dynamics (RQ2)

For RQ2, linear multivariate regressions are estimated at the monthly level. For each model x dimension regression (e.g., xlm-roberta_shared x Big Five Conscientiousness), email labels are aggregated to a monthly index on the [-100, 100] scale (as defined above), which serves as the dependent variable. Independent variables are the generalized personal and environmental characteristics described in Contextual variables section. The email archive spans January 2008 March 2024; the work-activity series begin in October 2009. Months with no emails of at least 10 words are omitted, yielding 167 monthly observations prior to outlier handling. Period variables were coded from dated records before regression modeling and without inspecting model-specific outcome series; Appendix B summarizes the coding rules. The participant-author drafted the generalized variable set from those dated records and froze the set before the regressions were estimated.

The regression pipeline included multicollinearity checks, outlier screening, HAC-robust standard errors, and Benjamini-Hochberg false-discovery-rate control within model families. HAC-robust standard errors were used because the dependent variables are monthly language indices and may contain autocorrelated and heteroskedastic residuals. The main specification was kept linear and comparable across model x dimension combinations because the purpose was descriptive within-person comparison across many theory dimensions and model families, not forecasting a single outcome series. To examine whether this baseline specification was sensitive to additional time-series issues, we conducted robustness checks for trend, spline trend, annual seasonality, lagged dependent variables, AR(1) structure, HAC bandwidth, outlier handling, multicollinearity, sparse signal coverage, signal weighting, lead/lag timing of focal periods, and random-window placebo effects. These checks are summarized in Appendix C, Table C1. The robustness analyses do not make the design causal, but they reduce the risk that the reported temporal patterns are artifacts of autocorrelation, secular drift, annual cycles, sparse monthly signal, or arbitrary event-window timing.

Additional analytical details are provided in Appendix C.

## Results

### Inter rater agreement

Across 2,000 emails, GPT 3.5 Turbo (few shot) and the agentic pipeline exhibit fair concordance (mean Cohen’s κ = 0.22, range = 0.02-0.64; mean Fleiss’ κ = 0.09, range = 0.50-0.64). Within model consistency is higher: for GPT 3.5, κ between the evaluator and the zero /few shot raters is 0.500.84 (moderate-substantial), consistent with prompt sensitivity; for the AI agentic system, κ between two rater agents is 0.80-0.90, and between the reflection and judge agents 0.971.00, indicating near identity at those stages. It also indicates that there is no benefit from adding the judge agent and cross-theory perspective, as judge ratings closely reflect the theory specific reflection ratings.

**Figure 2:**
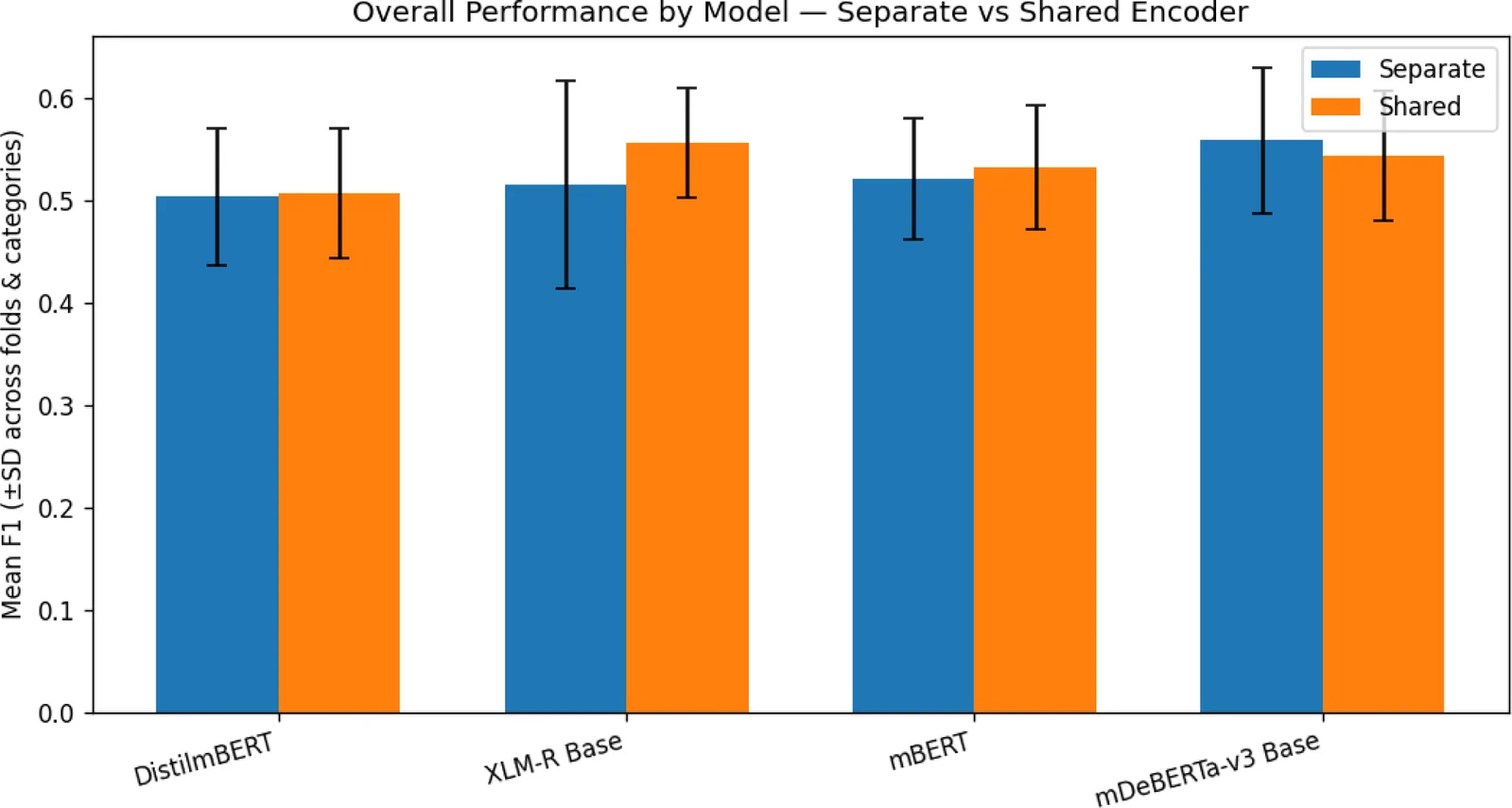
Mean F1 scores and standard deviations across five folds and categories, shared vs. separate encoder.

**Figure 3:**
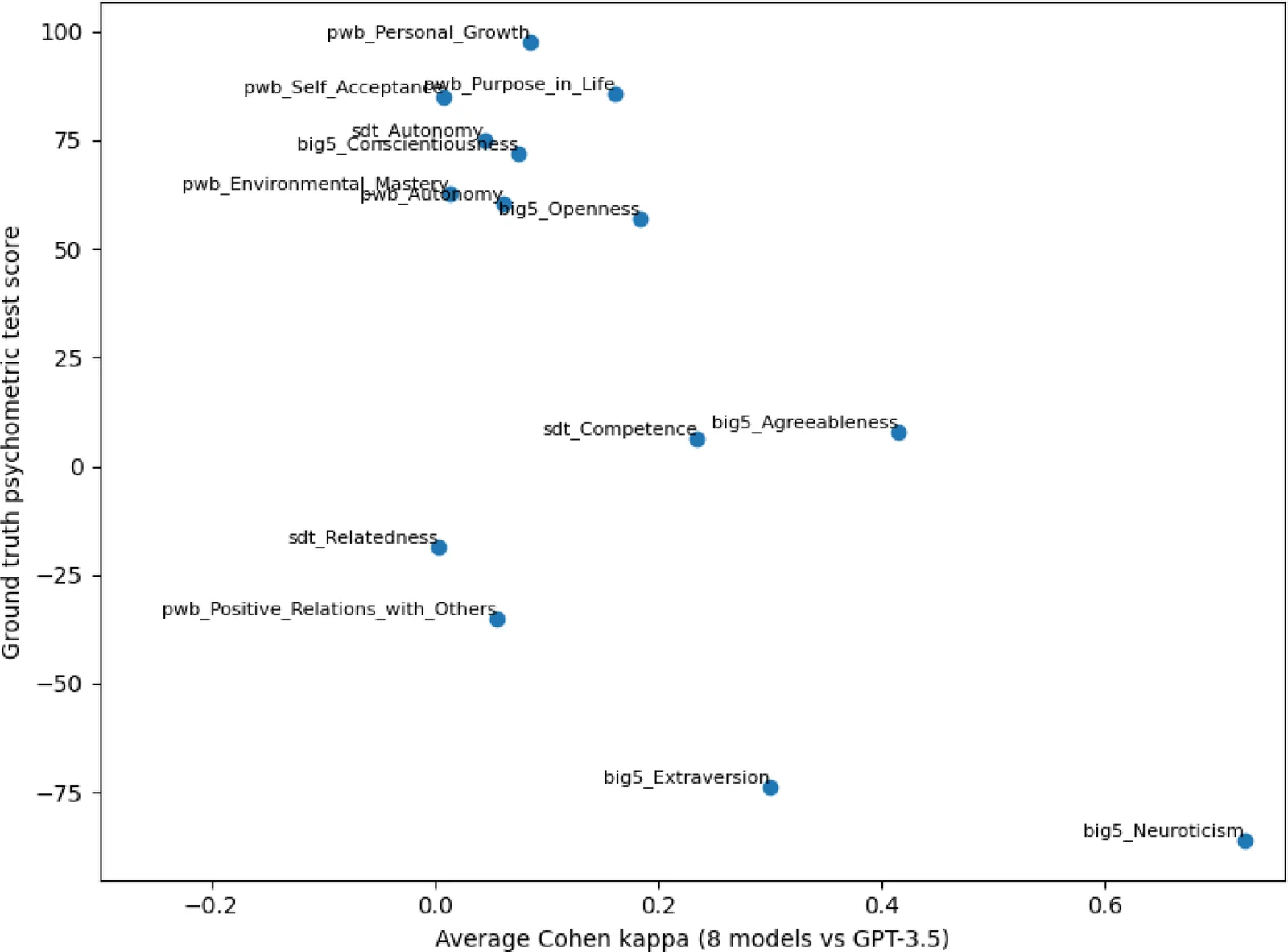
Negative relationship between the average Cohen kappa calculated for eight transformer models per dimension and the rescaled POMP score based on the participant-author’s self-report reference profile (Pearson correlation = -0.636).

Four multilingual transformers fine-tuned on judge labels (5 fold cross validation, 14 dimensions) achieve macro F1 patterns in which shared encoder variants are marginally superior overall, with xlm roberta base strongest; however, mdeberta v3 base favors separate encoders and the difference is not statistically significant (Figure 2). Replicating against GPT 3.5 over all eight fine-tuned models yields κ ≈ 0.0-0.7 across dimensions; agreement tends to be higher for traits with lower psychometric standing, except for relationship constructs (Figure 3).

### Classification of all emails

**Figure 4:**
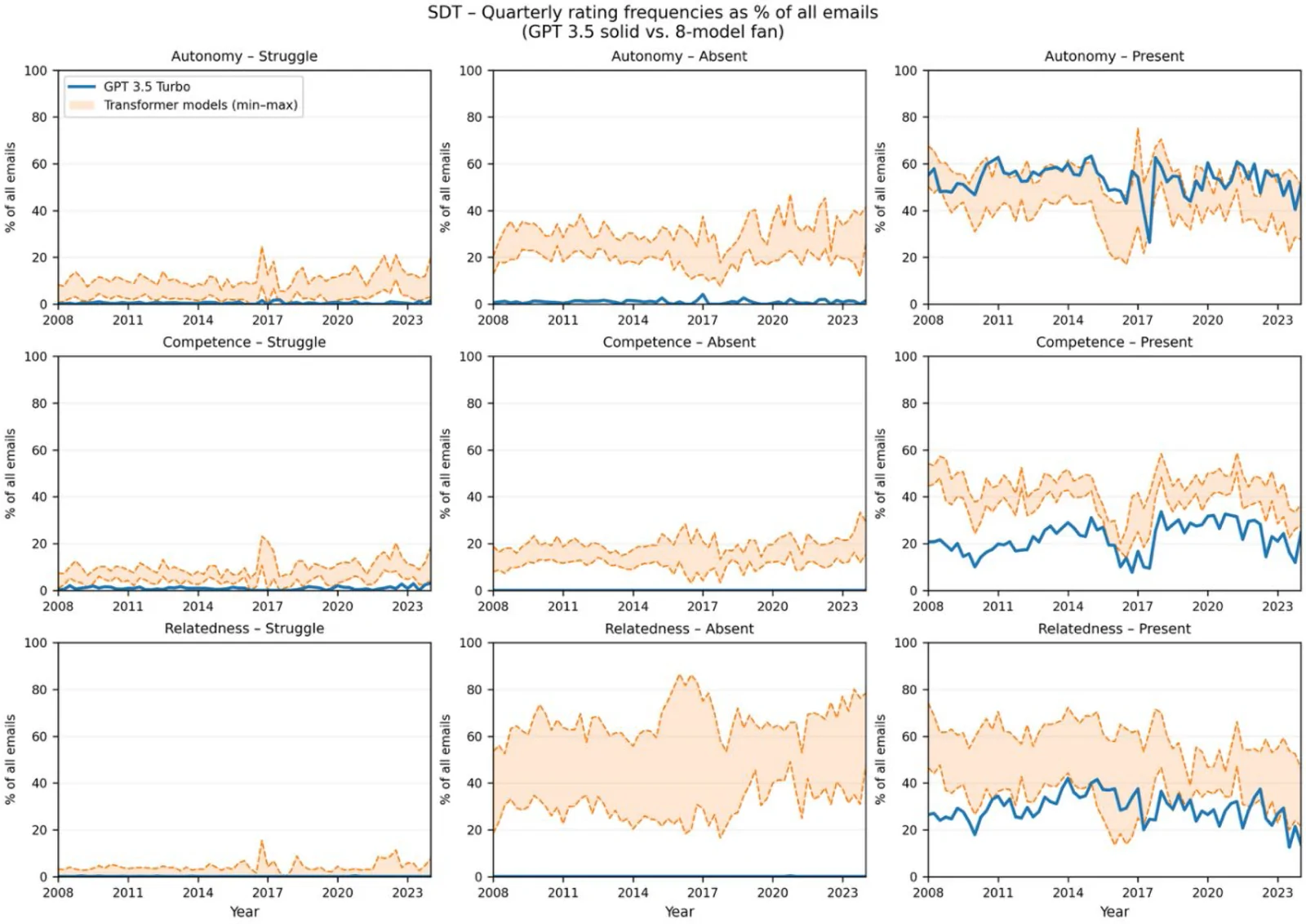
Email classification for SDT dimensions: GPT 3.5 Turbo (solid line) and eight smaller transformer models (min-max fan).

**Figure 5:**
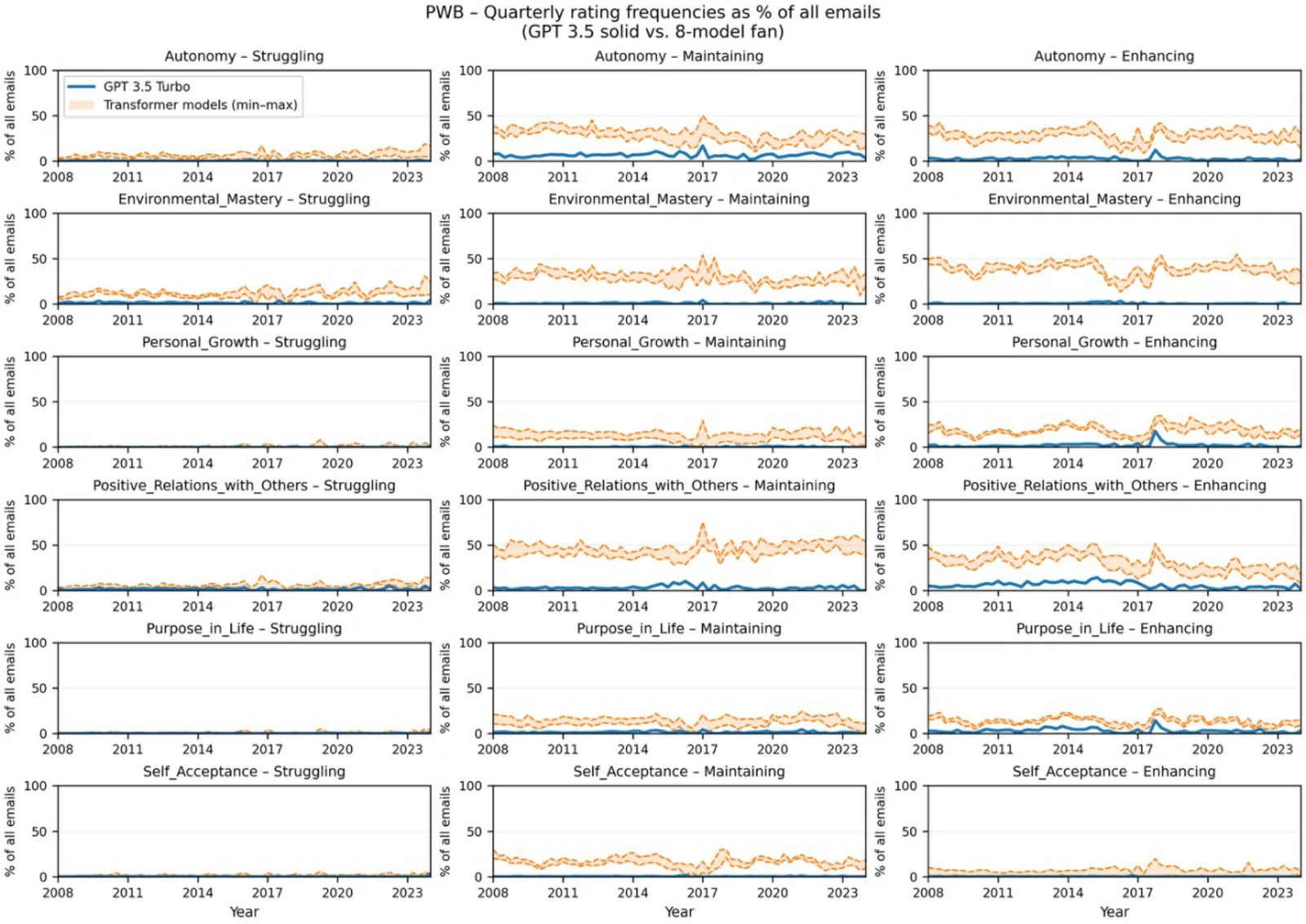
Email classification for PWB dimensions: GPT 3.5 Turbo (solid line) and eight smaller transformer models (min-max fan).

**Figure 6:**
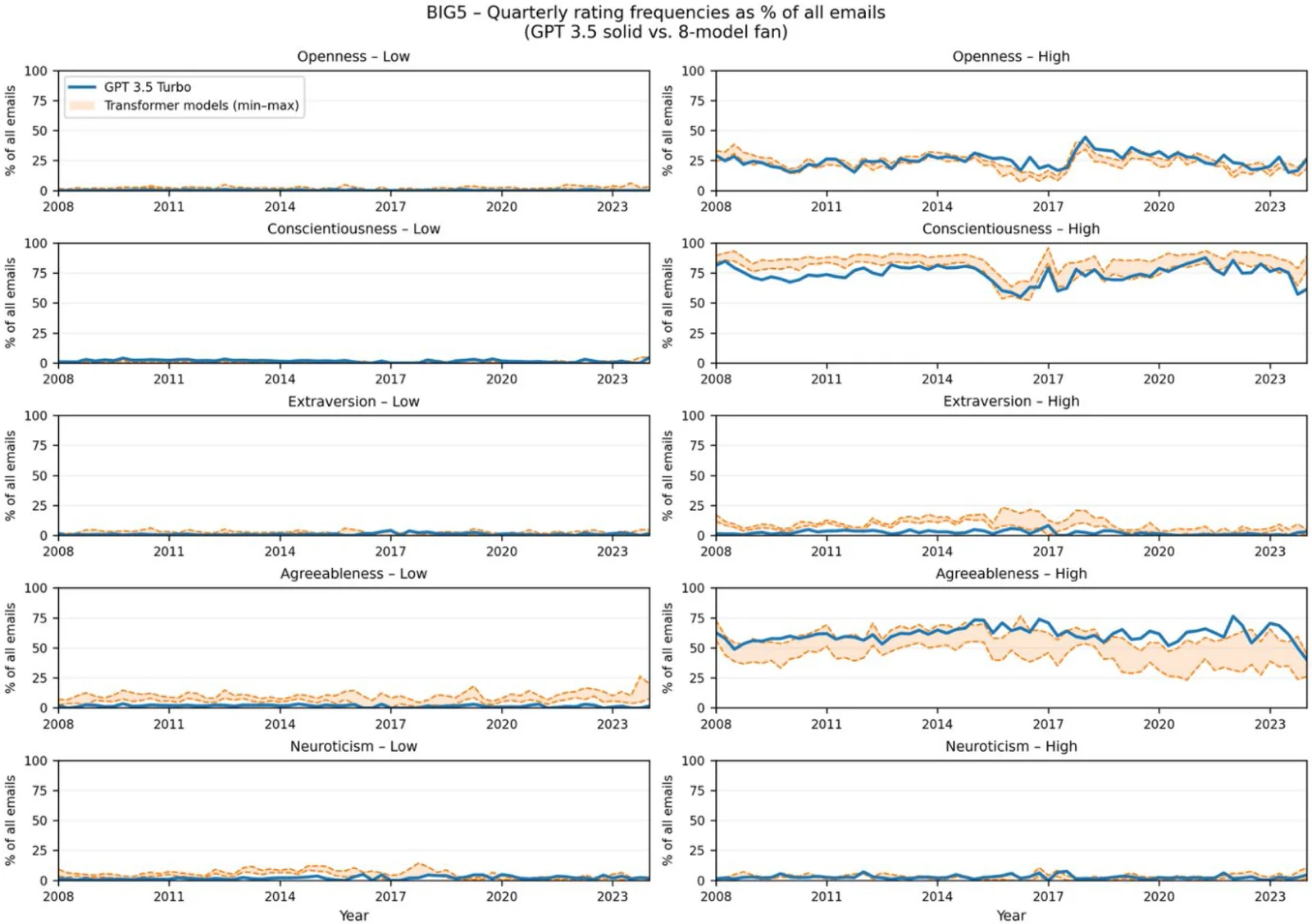
Email classification for Big Five traits: GPT 3.5 Turbo (solid line) and eight smaller transformer models (min-max fan).

Figures 4-6 display, by quarter, the share of all emails assigned to each label for GPT 3.5 Turbo (few shot rater) and for eight fine tuned transformers (shown as a min-max band); Not Applicable/None labels are omitted from the plots. Across dimensions, positive labels are sometimes higher for GPT 3.5 and sometimes for transformers, whereas neutral/negative labels are consistently higher for transformers, consistent with their lower NA rate (especially for PWB, where GPT 3.5 rarely assigns non NA labels). Despite these level differences, the longitudinal profiles of positive labels co vary across methods (typical *r*≈0.40-0.80), indicating partial agreement in change over time even when absolute prevalence differs. This temporal co movement characteristic is further evaluated with associations with life events in section reporting Study 2 results.

The figures should be read as prevalence-over-time plots. The blue line gives the GPT-3.5 few-shot rater; the orange band gives the minimum-to-maximum range across the eight transformer students. Overlap between the line and the band indicates similar prevalence estimates, whereas parallel movement with different levels indicates agreement in temporal pattern but disagreement in absolute label frequency. Because the archive is a professional email archive, changes over time are interpreted as changes in expressed language, not as direct changes in underlying traits.

### Study 1 - within-person profile correspondence (RQ1)

**Figure 7:**
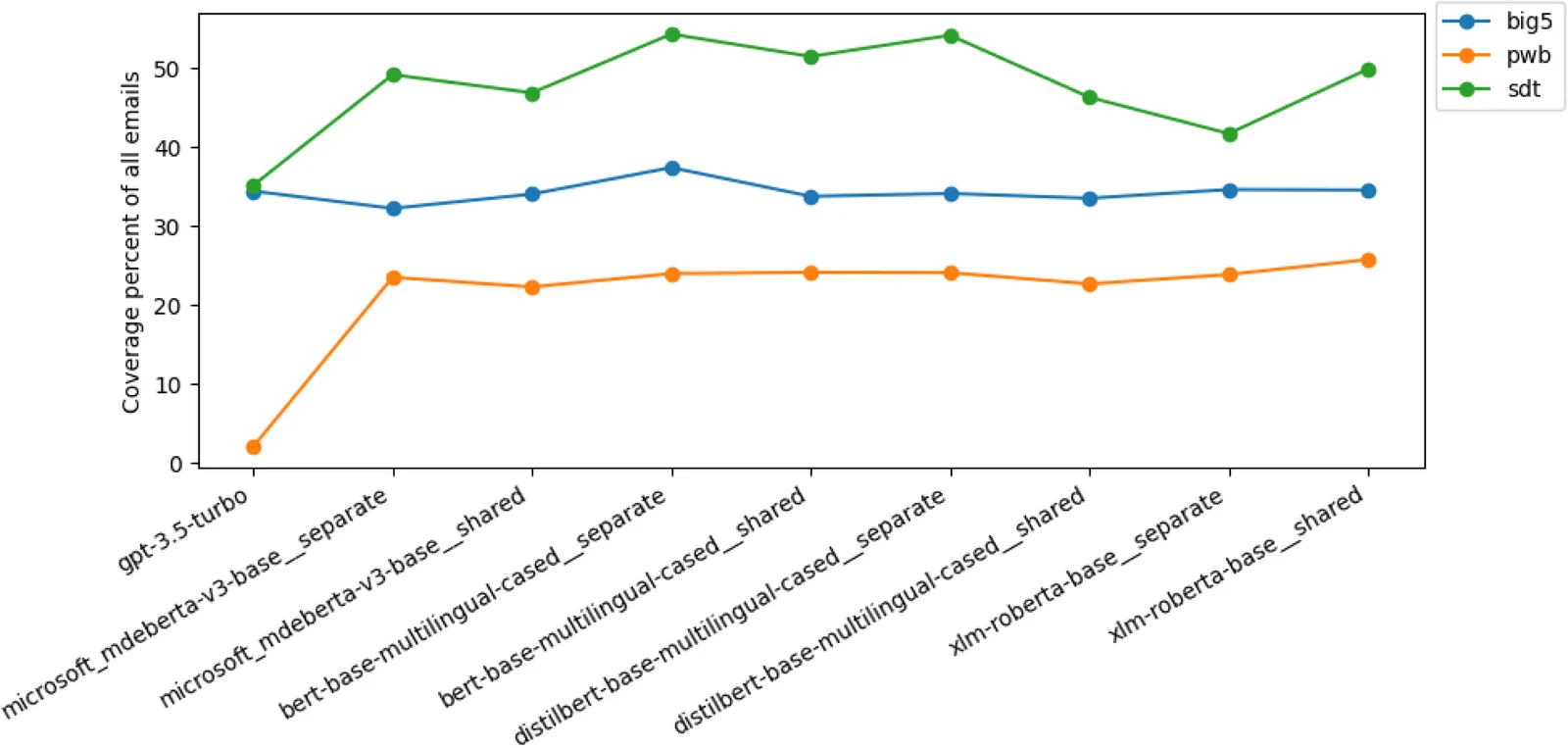
Email coverage by theory, average percentage of emails rated as positive or negative trait.

**Figure 8:**
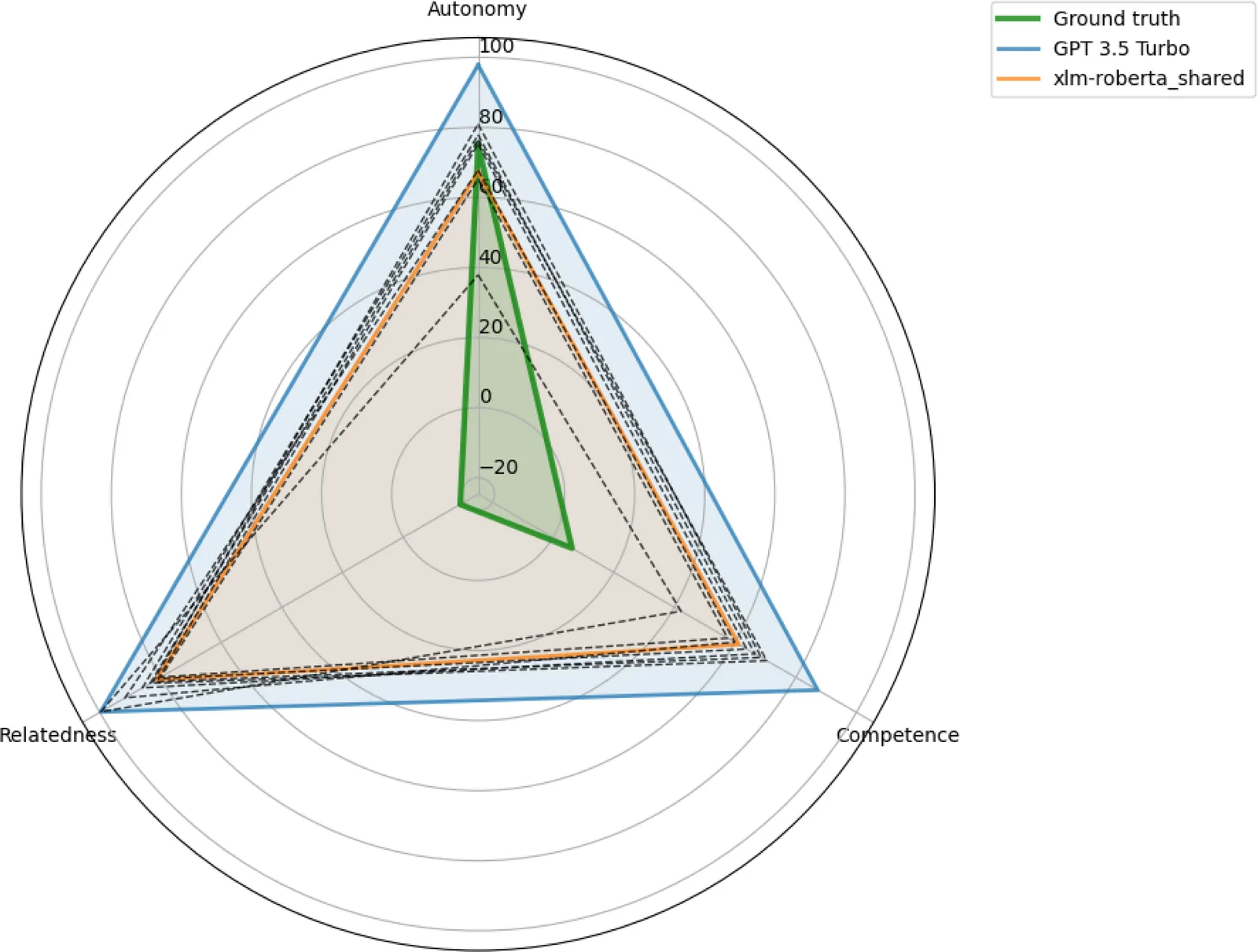
Comparison of self-report reference profiles with email ratings by various AI models, SDT. Green = self-report reference; blue/orange = selected model profiles; grey dashed lines = other transformer models.

**Figure 9:**
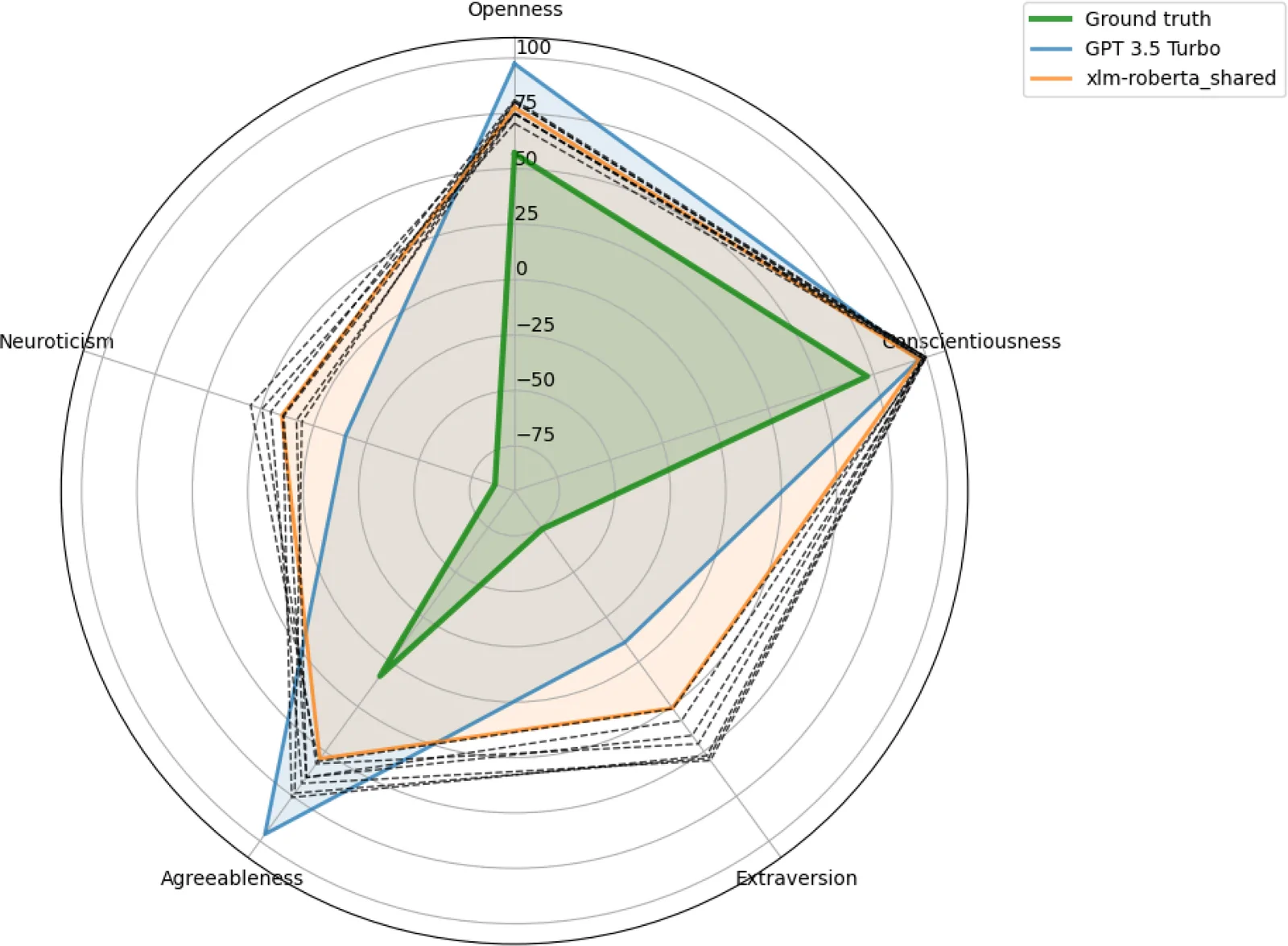
Comparison of self-report reference profiles with email ratings by various AI models, Big Five. Green = self-report reference; blue/orange = selected model profiles; grey dashed lines = other transformer models.

**Figure 10:**
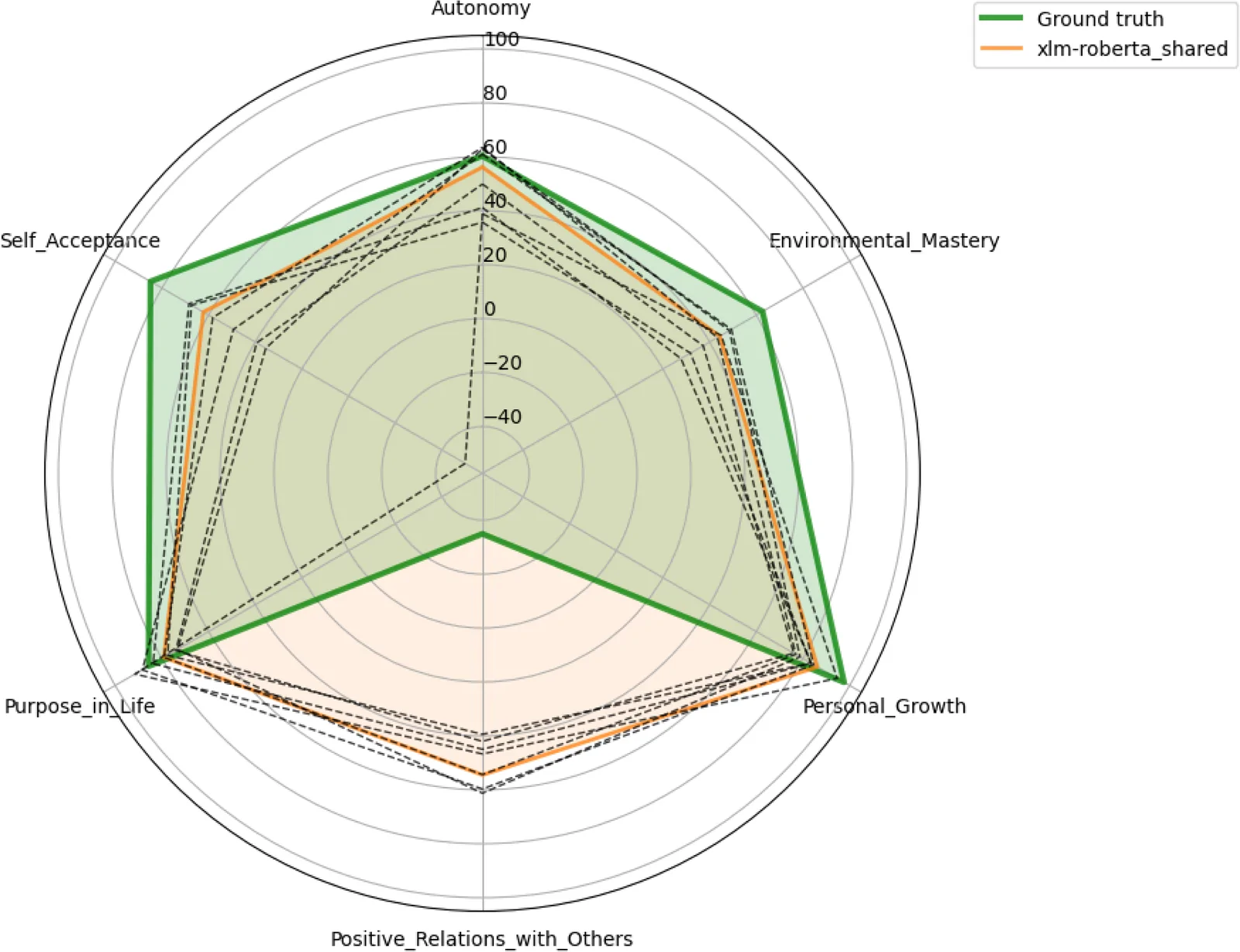
Comparison of self-report reference profiles with email ratings by various AI models, PWB. Green = self-report reference; orange = selected transformer profile; grey dashed lines = other transformer models.

Figures 8-10 are presented as radar charts that compare AI-based email profiles with self-report reference profiles. The best-performing transformer – xlm-roberta_shared, selected by the lowest across-dimension RMSE – is shown as a labeled solid line; other models are displayed as dashed overlays for context. For PWB, GPT-3.5 Turbo is intentionally omitted because non-NA coverage is insufficient to yield stable estimates (see Figure 7).

In these radar charts, the green polygon is the self-report reference profile, the blue line is GPT-3.5 when coverage is sufficient, the orange line is the selected transformer, and grey dashed lines show the remaining transformer models. The reader should focus on the shape of the profile--which dimensions are relatively high or low within the person--rather than the exact radial distance of any single point.

All radar charts employ a common [-100, 100] scale. The self-report reference profiles are obtained by mapping questionnaire scores to POMP (0-100) and then recentering/ rescaling to the [-100, 100] axis, whereas the model-based indices reflect the balance of positive vs. negative labels for each dimension. Consequently, absolute levels are not directly comparable. The radar plots are intended to assess broad within-case shape resemblance, not scale equivalence, between questionnaire profiles and email-derived indices.

Across many dimensions, the email-based and self-report reference profiles exhibit broadly similar shapes, yet several systematic differences are apparent. Relatedness (SDT) and Positive Relations with Others (PWB) are elevated in the email register relative to self-report. Human language exhibits a linguistic positivity bias across corpora (Dodds et al., 2015), and professional correspondence frequently amplifies affiliative and polite expressions. When such markers are mapped to relatedness/warmth, email-based indices can exceed self-report baselines without implying greater lived closeness in the person as a whole.

A second recurrent difference is the register-aligned elevation of Extraversion and Neuroticism relative to the self-report reference profiles, which indicate low standing on these traits. Email is inherently interactional - coordinating meetings, persuading, thanking – so it is rich in social and positive-affect language that correlates with Extraversion in open-vocabulary analyses (Schwartz et al., 2013). Seniorrole discourse can further accentuate an expressive, agentic style, producing extraversion-congruent signals even when self-reports indicate low Extraversion. By contrast, the email corpus, which is dominated by work-related communication, contains substantial problem- and risk-focused wording that overlaps with negative-affect lexicon typically associated with Neuroticism in open-domain corpora. In professional registers, these cues often reflect task exigencies rather than enduring distress, which should be interpreted as a register effect of task-bound problem framing, not evidence of higher trait Neuroticism.

### Study 2 - within-person temporal dynamics (RQ2)

**Figure 11:**
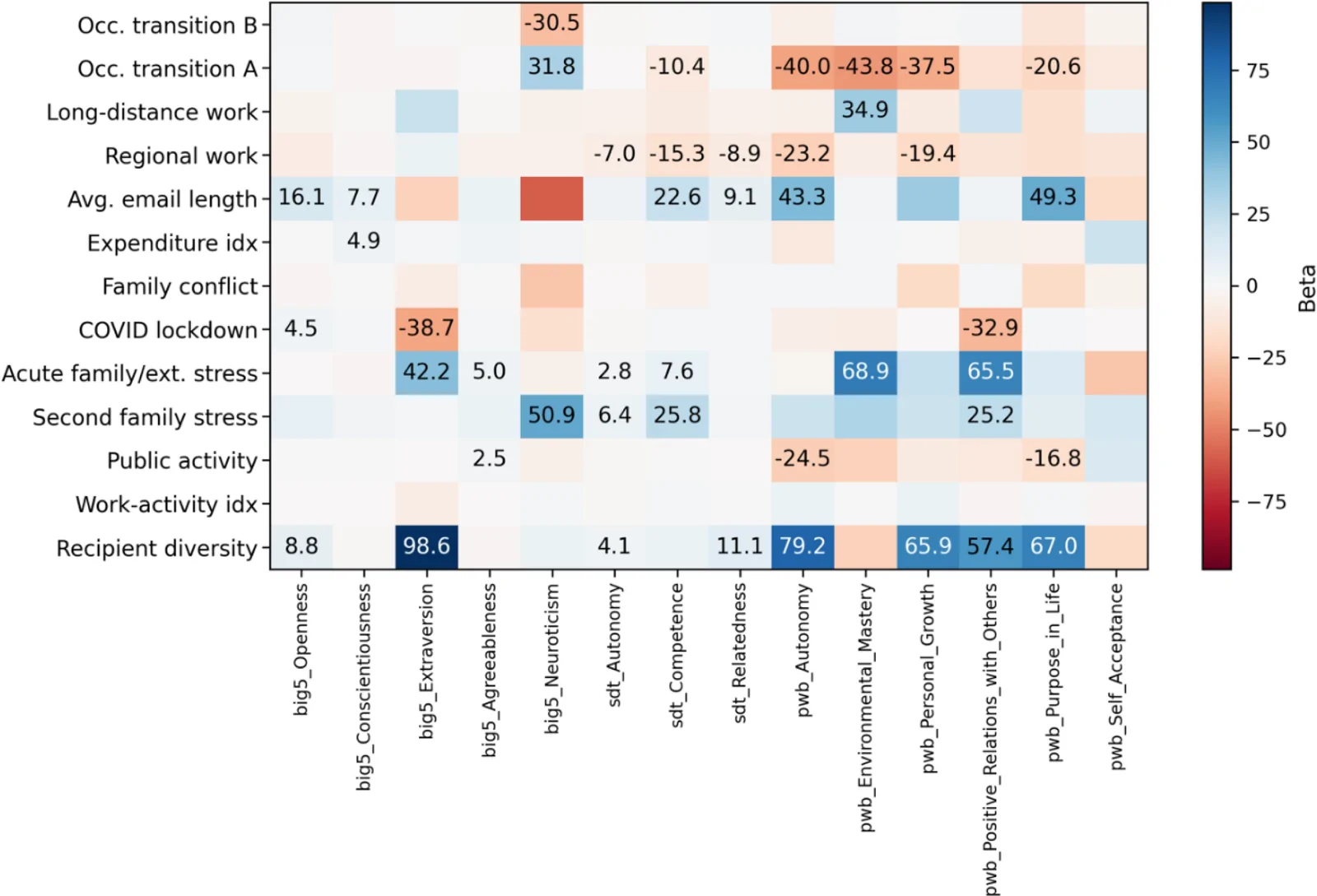
Regression results for GPT-3.5 Turbo email ratings. Blue cells indicate positive coefficients, red cells negative coefficients; printed values denote FDR-significant associations.

**Figure 12:**
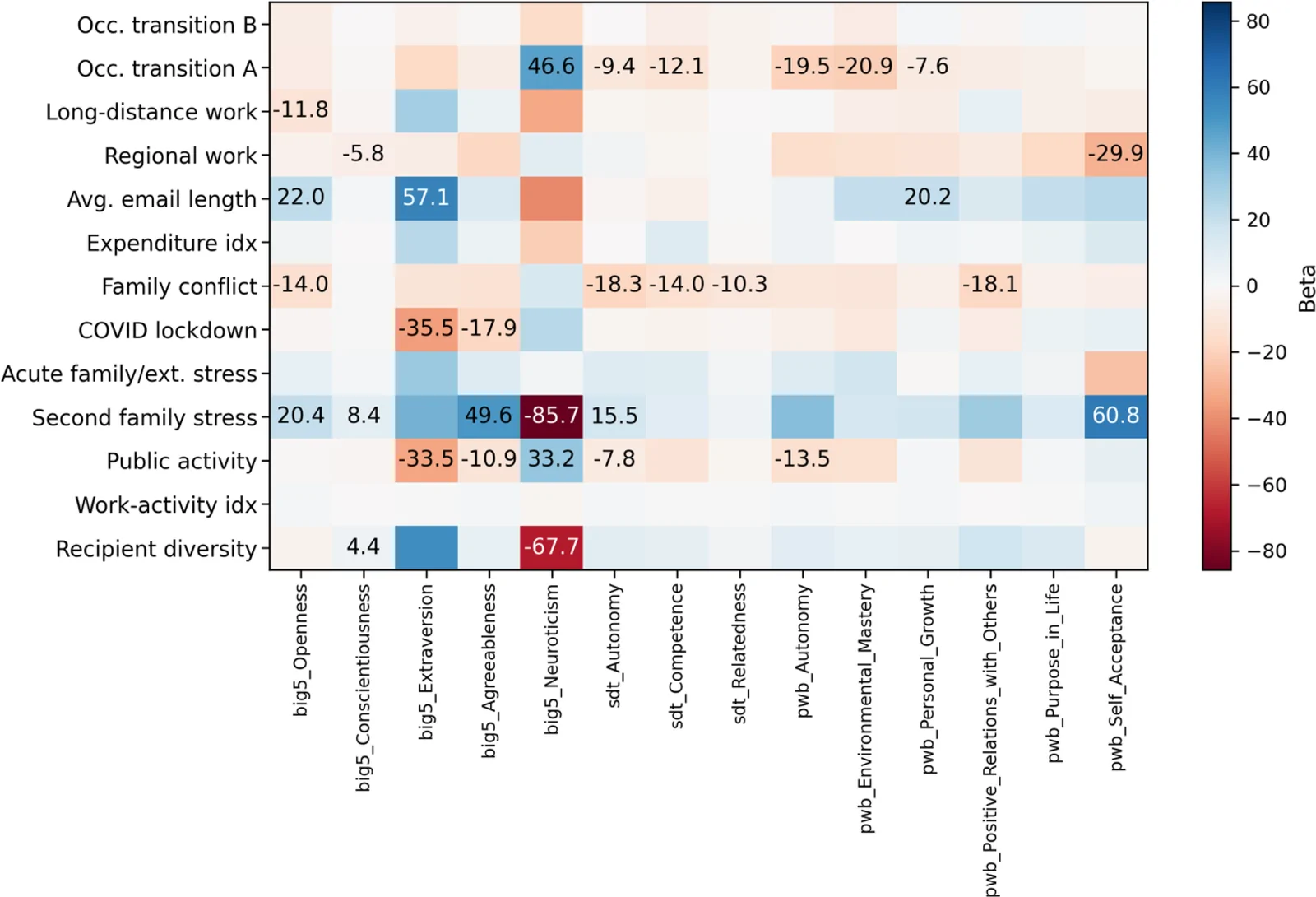
Regression results for email ratings by transformer/agentic AI model (xlm-roberta-shared fine-tuned on judge AI agent labels). Blue cells indicate positive coefficients, red cells negative coefficients; printed values denote FDR-significant associations.

Figures 11-12 display heatmaps summarizing monthly regressions of email-based indices on personal and environmental covariates. Each column corresponds to one model x dimension regression; rows list predictors (intercept omitted). Cells report unstandardized coefficients where FDRadjusted *p* < 0.05; non-significant cells are only colored according to scale.

Thus, each heatmap should be read row by row: a colored cell marks the estimated within-person association between a contextual period or covariate and one email-language dimension. Blank or unnumbered cells are not interpreted as meaningful findings, even when lightly colored by the common scale.

Consistent associations are observed between three stressful periods and adverse shifts in text-derived indices. During major occupational transition A – characterized by intensive, revenue-oriented professional demands – both modeling approaches indicate a rise in Neuroticism coupled with declines in Autonomy, Competence, Environmental Mastery, and Personal Growth; Purpose in Life also declines in the GPT-3.5 specification. These patterns align with stress- and control-related interpretations of the period.

A broadly similar profile appears around the high-visibility public activity period, which is associated with increased Neuroticism and lower levels on several positive dimensions; however, model-specific differences emerge (including disagreement on Agreeableness), indicating that the linguistic manifestations of this period are captured differently by GPT-3.5 and the transformer/agentic AI models.

The emotionally exhausting family conflict period coincides with a further decline in already low Relatedness and with reductions in Openness, Autonomy, and Competence that are captured in the transformer/agentic-AI-based regressions but not uniformly by GPT-3.5. These model-specific findings suggest either genuine theory- or register-sensitive signals or differences in how models weigh problem-focused, formal correspondence.

Interpretation of these associations should consider several design features. The analyses are observational and subject-specific; coefficients quantify contemporaneous covariation within one person’s archive, not causal impact. HACrobust regression accounts for autocorrelation in the standard errors but does not model all possible forms of temporal dependence. The added robustness checks examine trend, seasonality, lagged dependence, AR(1) structure, outlier influence, multicollinearity, sparse signal coverage, lead/lag timing, and random-window placebo effects (Appendix C, Table C1). Multi-month periods may still exhibit anticipatory or lagged shifts. The added lead/lag checks therefore treat timing as exploratory rather than causal: they indicate whether selected focal-period associations appear contemporaneously, before, or after the coded period, but they do not establish temporal ordering as a causal mechanism. Taken together, the heatmaps indicate that major occupational and personal periods are systematically associated with theoretically coherent changes in email-based personality-related language within this case, but they should not be read as causal estimates.

## Discussion and Limitations

This paper presents a subject-specific, person-oriented analysis of personality-related language in a large archive of mostly professional emails. Across methods, the study finds moderate agreement in levels and substantial co-movement over time, indicating that different families of models – GPT-3.5 with few-shot prompting and a GPT-4.1-based teacher-student pipeline – recover partly convergent dynamics within one person’s archive.

Viewed as a whole rather than as isolated variables, the case is characterized by strong task orientation and self-regulatory language, relatively low self-reported Extraversion and Neuroticism, and a professional register that inflates affiliative and socially expressive cues in email. The clearest within-person downturns cluster around periods of constrained control, acute stress, and unusually public or occupational exposure. This whole-pattern reading is more informative for person-oriented purposes than any single trait estimate because it shows how needs, broad traits, and well-being move together within the same person.

The study also clarifies where LLM-assisted coding may systematically over- or under-emphasize constructs when operating in a domain dominated by work email. Relative to self-report benchmarks, model-based ratings tend to elevate Relatedness/Positive Relations and Extraversion and to over-flag Neuroticism as a function of register (politeness, coordination, risk signaling), not necessarily as model error. Two domain forces plausibly explain this: (1) a linguistic positivity bias that characterizes many corpora and is amplified in professional communication, and (2) a role-specific requirement to signal problems, urgency, and responsibility, which can resemble negative affect or vigilance in text.

Method comparisons are useful here primarily as robustness checks. The main point is that the central subject-specific profile and temporal covariation were visible across several workflows, while reflection improved stability and the added judge stage contributed little incremental signal. For person-oriented repeated-measurement designs, that finding suggests that simpler workflows may be sufficient if they preserve the same within-person pattern.

The measurement strategy – balancing positive and negative labels on a common [-100, 100] range, and comparing profile shapes rather than absolute levels to POMP-scaled self-report reference profiles – offers a practical way to triangulate one person’s email-derived signals with self-reports when scales differ. In this case, lower agreement tended to cluster on dimensions that were weakly expressed in a professional register, underscoring that idiographic interpretation must consider both construct meaning and communicative context.

### Implications for personality science and practice

This is a subject-specific, mostly professional-email study of within-person profile correspondence and temporal dynamics. Findings are not intended for between-person generalization or human-parity claims. The design triangulates AI-derived email indices with self-report benchmarks and period-linked longitudinal models, enabling idiographic interpretation of one person’s profile and shifts across time.

The study advances the conversation on how digital traces can support person-oriented research on within-person dynamics. The results suggest a pragmatic division of labor: self-report inventories remain the primary reference for trait baselines, while language-based indices may be strongest as state-sensitive barometers of contextualized functioning (e.g., shifts in autonomy-relevant language when job demands threaten control). In applied settings this implies using text-based measures as early-warning signals and process measures, not as stand-alone diagnostic replacements.

The automated nature of the coding also suggests a possible extension to multiple subject-specific archives. Such work would not have to abandon the person-oriented logic by averaging all persons into one population model. Instead, each person’s profile and time-series model could first be interpreted idiographically, and then cases could be compared for recurring configurations, contrasts, or dynamic typologies. This would allow limited generalization across persons while preserving the subject-specific basis of the analysis.

Methodologically, the evidence here suggests that reflection mechanisms can improve consistency in theory-guided text coding and that domain-tuned small models distilled from stronger teachers can outperform prompt-only LLMs for specialized classification (Pangakis & Wolken, 2024; Hu et al., 2024). For person-oriented research, however, the practical implication is not to maximize model complexity for its own sake, but to choose a workflow that is transparent, affordable, and stable enough for repeated within-person measurement.

### Limitations

The corpus is from one participant-author, and the emails are predominantly professional. Findings may not extend to other roles, cultures, or private communications; role norms likely shape the positivity/sociality signatures discussed above. Replication across additional subject-specific archives with diverse occupations, languages, and platforms would clarify which features are person-specific and which are domain-specific.

The author-as-subject design has both advantages and risks. Its advantage is access to a long private archive, dated contextual records, and interpretive knowledge that would be difficult to obtain from an external participant. Its risk is that the same person who generated the data also selected the contextual interpretation and wrote the analysis. The study addresses this risk by using pre-specified generalized contextual variables, de-identification procedures, cross-model comparisons, and explicit limitation of claims to one case, but the possibility of selective contextualization cannot be eliminated.

The radar-chart profile-correspondence strategy mitigates scale differences but does not solve temporal mismatch or common-method variance. The use of a range-based profile display is also consistent with recent person-oriented guidance warning that *z*-scores can distort profile interpretation and obscure endorsement meaning in graphical comparisons (Moeller, 2025). Moreover, the self-report reference profiles were assembled from mixed-source instruments, and the PWB coverage by GPT-3.5 was limited (many NA labels), constraining inferences for that theory.

A further limitation concerns the Big Five framework itself. The Big Five taxonomy was developed mainly from between-person covariation in descriptors and is not designed primarily to capture individuality or within-person organization. It remains useful here as a broad vocabulary for traitrelated language, but it should not be treated as a complete person-oriented account of the subject. Future work could combine Big Five coding with more idiographic approaches such as personal goals, personal projects, narrative identity, or role-specific motives.

Distilled transformers inherit whatever biases and blind spots are present in the teacher (e.g., over-reading politeness as Relatedness). While reflection and judging can reduce noise, they may also entrench shared misconceptions (Fu et al., 2023; Li et al., 2024). Independent human coding would be a useful extension in future multi-author studies, but the present subject-specific design instead relies on triangulation among self-report benchmarks, cross-model convergence, and period-linked temporal patterns.

Within-model kappa for GPT-3.5 varied by prompt configuration, underscoring prompt sensitivity and the importance of example selection. Label taxonomies differ across theories (e.g., presence/struggle vs. high/low vs. enhancing/maintaining) and collapsing to a single index (positive vs. negative) risks information loss.

Finally, the degree to which personality traits, needs, or well-being are expressed in email may itself be person-specific. Some individuals may disclose stress, conflict, or need frustration directly; others may write in a controlled professional register even during difficult periods. Neuroticism is a useful example: worry or emotional instability may be visible in text only when the writer chooses to express it, or when task demands require problem-focused wording. Absence of a signal in email should therefore not be interpreted as absence of the psychological characteristic in the person.

### Conclusions

This research shows that everyday professional emails can carry psychologically meaningful signals that can be interpreted within three major frameworks – Self-Determination Theory, the Big Five, and Psychological Well-Being – using prompted LLMs and teacher-student pipelines. In this 16year, 25,780-email archive, different modeling families show moderate cross-method agreement yet strong longitudinal co-movement, and the resulting indices shift across major occupational and personal periods in theory-coherent ways. The contribution is subject-specific: it demonstrates how one person’s de-identified language archive can be used to describe profile shape and temporal dynamics, while also showing how professional register shapes the signals that models recover. Self-reports remain the primary reference for trait baselines, and between-person generalization is not warranted.

## Data Availability

Raw emails cannot be shared because they are private correspondence. Code, condensed prompts, and derived replication materials are available in the associated Zenodo replication archive https://zenodo.org/records/21071665; raw text and direct identifiers are excluded.

## Action editor

Lars-Gunnar Lundh served as action editor for this article.

## Open access

This article is distributed under the terms of the Creative Commons Attribution 4.0 International License (http://creativecommons.org/licenses/by/4.0/), which permits unrestricted use, distribution, and reproduction in any medium, provided you give appropriate credit to the original author(s) and the source, provide a link to the Creative Commons license, and indicate if changes were made.

## Appendix A

### Ethics statement and data governance

This article reports a subject-specific self-study. The sole author was also the sole participant and source of the archival email and self-report data analyzed in the manuscript. On 3 March 2026, the Ethics and Anti-Discrimination Committee of Vistula University approved the project from an ethical standpoint for publication. The signed approval form did not state a reference number. The approval was signed on behalf of the committee by Dr Davut Han Aslan, chairman. The participant-author provided written informed consent for secure archive retrieval, de-identification, analysis, and publication of aggregate findings.

### Data minimization and de-identification

Only text authored by the participant-author was analyzed. Quoted and forwarded material from other people was excluded at preprocessing. Direct identifiers, including email addresses, were removed or masked in working files, and the article reports only aggregate statistics, figures, and generalized contextual descriptions. No verbatim email text is reproduced in the manuscript or supplement.

### Residual third-party risk

Professional correspondence can still contain residual references to other people or organizations even after filtering. To mitigate this risk, the project uses de-identification, broad contextual labeling, suppression of verbatim quotations, and non-release of raw email text. The study does not make person-level claims about any third party.

### Data security and sharing

De-identified working files were stored on access-restricted devices under the control of the author. Raw emails are not publicly shared because they are private correspondence. Only derived materials consistent with consent, privacy protection, and journal requirements may be shared. Direct identifiers and raw message text are excluded from any such sharing.

## Appendix B

### Contextual variables, pre-specification, and minimal contextual summary

#### Minimal contextual summary

The participant-author worked primarily in senior professional and executive roles in a European context. The archive spans 2008-2024 and is predominantly professional correspondence. It includes periods of work abroad, occupational transition, unusually public-facing activity, and family stress. These contextual descriptors are used only to interpret within-person patterns over time and not for between-person inference.

#### Pre-specification principle

Contextual variables were drafted by the participant-author from dated administrative, calendar, employment, and personal records before inspection of the language trajectories. The generalized variable set was then frozen before any model-specific regressions were run. The same generalized variable set was then applied across all model families. To reduce identifiability, several originally distinct real-world events were collapsed into broader categories.

**Table A1:** Contextual variables, coding rules, and source/timing.

Generalized variable/period	Coding rule	Source and timing
Work-activity index	Combined monthly work-related receipts, normalized to the period-wide average.	Monthly administrative records; fixed before modeling.
Personal expenditure index	Monthly card expenditures normalized to [0,1].	Monthly financial records; fixed before modeling.
Long-distance work assignment	1 during months with an extended work assignment abroad; otherwise 0.	Calendar/employment records; coded before trajectory inspection.
Regional work assignment	1 during months with a shorter-distance work assignment abroad; otherwise 0.	Calendar/employment records; coded before trajectory inspection.
Acute family/external stress period	1 in the focal month, 0.75 in the next month, 0.5 in the following month.	Dated personal records; coded before trajectory inspection.
Second acute family stress period	1 in the focal month, 0.75 in the next month, 0.5 in the following month.	Dated personal records; coded before trajectory inspection.
Family conflict period	1 during months of an emotionally demanding family conflict; otherwise 0.	Dated personal records; coded before trajectory inspection.
Major occupational transition A	1 during a high-intensity senior professional transition; otherwise 0.	Employment records; coded before trajectory inspection.
Major occupational transition B	1 during a senior executive transition in a technology-focused organization; otherwise 0.	Employment records; coded before trajectory inspection.
High-visibility public activity period	1 during months with unusually public-facing professional activity; otherwise 0.	Calendar/public records; coded before trajectory inspection.
COVID lockdown	1 during months with strict lockdown conditions; otherwise 0.	Public dates; fixed in advance.
Recipient diversity	Monthly count of unique recipients, normalized to [0,1].	Computed from email metadata after preprocessing.
Average email length	Monthly mean word count per authored email, normalized to [0,1].	Computed from the processed corpus.

These variables serve interpretive and control functions within a single-case time series. They are not intended as population-level predictors and should not be read as claims about general causal effects.

## Appendix C

### Additional analytical details

#### Email-level balance index

For each theory dimension, monthly scores are derived from the balance of positive versus negative email labels on a common [-100, 100] scale. The practical scoring rule used in the manuscript is a Jeffreys-prior-stabilized balance index: Index = 100 x [((positive + 0.5) - (negative + 0.5)) / ((positive + 0.5) + (negative + 0.5))].

#### Self-report reference profiles

For SDT and PWB, raw subscales were transformed to Percent of Maximum Possible (POMP; Cohen et al., 1999); for Big Five, the available OpenPsychometrics (https://openpsychometrics.org/tests/IPIP-BFFM/) percentile outputs were linearly mapped to the common [-100, 100] profile axis because item-level responses were not retained. POMP = 100 x [(observed score - minimum possible score) / (maximum possible score - minimum possible score)]. The resulting POMP values were then rescaled to the manuscript’s common plotting range: Rescaled profile = (2 x POMP) - 100.

This choice of a range-based transformation is also consistent with recent person-oriented recommendations to avoid z-standardization in profile displays when the goal is interpretability of within-person patterns (Moeller, 2025).

#### Monthly regressions

The unit of analysis for Study 2 is the month. For each model x dimension combination, the monthly balance index served as the dependent variable. Predictors included the generalized contextual variables described in Appendix B together with corpus-structure covariates such as recipient diversity and average email length.

The regression workflow included multicollinearity checks, residual diagnostics, outlier screening, HAC-robust standard errors, and false-discovery-rate control within model families. All interpretations remain observational and within-person.

#### Additional time-series robustness checks

To evaluate whether the monthly regression results were sensitive to additional time-series features, we conducted the checks summarized in Table C1. These checks address possible non-stationarity, annual cyclicity, lagged dynamics, outlier influence, sparse signal coverage, and arbitrary timing of contextual periods. They are used as sensitivity checks for descriptive within-person associations, not as separate forecasting models. Full results are available in the replication package: https://zenodo.org/records/21071665.

**Table C1:** Summary of added time-series robustness checks for Study 2.

Robustness check	Time-series issue addressed	Implementation, result, and use in manuscript
Time-series diagnostic screen	HAC robust errors alone do not show whether the monthly series contain trend, non-stationarity, cyclicity, or residual dependence.	For the 28 primary model x dimension series, the revision computed ADF/KPSS stationarity checks, residual ACF/Ljung-Box diagnostics, and joint tests for trend and seasonality. Diagnostics were mixed: trend evidence appeared in 14/28 series and seasonality evidence in 4/28 series. This supports using sensitivity checks rather than claiming that the series are free of time structure.
HAC standard errors and bandwidth sensitivity	Monthly observations can have autocorrelated and heteroskedastic residuals.	The primary monthly regressions use HAC/Newey-West standard errors. A widerbandwidth HAC sensitivity model was also estimated to check whether conclusions depended on the chosen autocorrelation window. HAC is treated as a transparent baseline correction, not as a complete time-series model.
Outlier influence	A small number of unusual months might drive regression coefficients.	Months with absolute z-scores above 3 on the monthly balance outcome were removed in the main pipeline, and a no-outlier-removal sensitivity model was also estimated. Across the 28 primary series, 67 months were removed in total, 23 series had at least one removed month, and no series lost more than 5 months.
Multicollinearity checks	Contextual predictors may overlap, making coefficients unstable.	Variance inflation factors were inspected for baseline and trend-seasonality specifications. No VIF exceeded 10. The largest values were Time scaled = 8.88 and Recipient diversity = 7.00 in the trend-seasonality model, so these predictors were retained but interpreted with monitoring.
Linear and spline trend sensitivity	Associations may reflect gradual secular drift rather than focal periods.	Models were re-estimated with linear time and spline time terms. Robust findings were required to retain a consistent direction across the sensitivity set, and the trend-and-seasonality-adjusted coefficient was used as the preferred adjusted sensitivity estimate.
Annual seasonality sensitivity	Monthly email language may follow annual or academicyear cycles.	Annual sine and cosine terms were added to estimate seasonality. The main adjusted specification combines trend and seasonality, so reported robust findings are not simply unadjusted calendar-cycle associations.
Lagged dependent-variable specification	Current monthly language may depend on the previous month, not only on current contextual periods.	A lagged-outcome model added the prior month’s balance index. The robustness table reports whether focal-period coefficients retained direction and significance when this autoregressive adjustment was included.
AR(1) alternative	Residual autocorrelation may remain after HAC correction.	A GLSAR AR(1) alternative with the trend-seasonality controls was estimated as an additional check. This was used as sensitivity evidence rather than as the primary model, because the goal was comparable description across many outcomes rather than forecasting one series.
Low-coverage and signalweighted models	Some monthly indices may be less reliable when few emails contain a construct signal.	Models were re-estimated after requiring at least one signal, at least five signals, and at least ten total emails per month; a weighted HAC model weighted months by signal count. This checks whether sparse months drive the reported patterns.
Lead/lag focalperiod tests	Relevant language changes may precede or follow a contextual period rather than occur only contemporaneously.	Focal period indicators were shifted two months earlier, one month earlier, current month, one month later, and two months later. These 560 coefficients are treated as exploratory timing diagnostics, not as new confirmatory claims.
Random-window placebo checks	Similar coefficients might appear for arbitrary periods of the same duration.	For 30 robust focal associations, 1,000 random windows of equal duration were sampled outside the true focal window. Ten associations had empirical two-sided p < .05, suggesting that a subset of focal-period effects was stronger than expected under random timing.
Multiple testing and reporting rule	A large grid of coefficients can produce isolated significant results.	Baseline coefficient families include Benjamini-Hochberg FDR-adjusted q-values, while sensitivity analyses emphasize directional stability across specifications. The full robust-results inventory contains 55 robust rows, but the manuscript narrates only selected theoretically interpretable patterns; the full robustness-result files are available in the replication materials.

Interpretation: these checks ask whether the main temporal patterns could be artifacts of month-to-month similarity, long-term drift, annual cycles, unusual months, sparse email signal, overlapping predictors, delayed or anticipatory timing, or arbitrary event windows. The answer is mixed but useful: the findings remain observational and non-causal, yet the most interpretable associations are not based on HAC alone; they are also screened against several alternative explanations.

## Appendix D

### Condensed model and prompt summary

The article retains several coding workflows as robustness checks on whether the same within-person pattern appears across different AI-assisted coding strategies. The main workflows are summarized below.

**Table:**

Workflow	Role in the study
GPT-3.5 zero-shot	Initial prompt-only baseline for theory-guided coding.
GPT-3.5 few-shot rater and evaluator	Prompted coding with two example sets, used as a practical LLM comparison and internal agreement check.
GPT-4.1 agentic teacher	Two raters plus reflection and judge stages, used as the strongest teacher workflow for detailed coding.
Distilled multilingual students	Compact transformer classifiers trained on teacher labels and used to test scalable replication of the within-person pattern (8 student models: separate-encoder and shared-encoder variants of microsoft/mdeberta-v3-base, bert-base-multilingual-cased, distilbert-base-multilingual-cased, and xlm-roberta-base).

### Theory-specific label sets

SDT labels: Present, Struggle, Absent, None. Big Five labels: High, Low, None. PWB labels: Enhancing, Struggling, Maintaining, Not Applicable. For longitudinal indices, neutral/non-informative states were excluded so that each monthly score reflected the balance of positive versus negative signals.

### What is intentionally omitted from the supplement

Full prompt dumps, long code excerpts, and implementation blocks are intentionally omitted from the supplement. They are not required for the reader to evaluate the person-oriented claims of the article and will be supplied separately as part of the external replication materials
